# Supplementary material for: Machine learning multi-omics analysis reveals cancer driver dysregulation in pan-cancer cell lines compared to primary tumors
Source: Commun Biol. 2022 Dec 13;5:1367. doi: 10.1038/s42003-022-04075-4 (PMC9747808; doi:10.1038/s42003-022-04075-4)
Supplement: Supplementary file 2 — Supplementary Information [file 42003_2022_4075_MOESM2_ESM.pdf]

Supplementary Figures

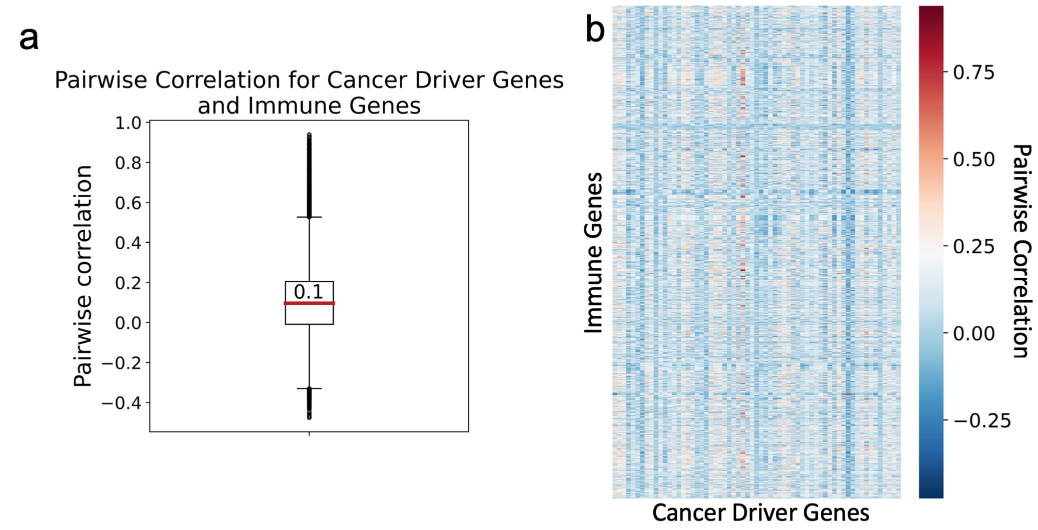

Supplementary Figure 1. Pairwise correlation of expression of immune genes and cancer driver genes. a) Pairwise correlation between the immune genes removed from the SVM analysis and the cancer driver genes identified by the SVM analysis (mean correlation=0.1). b) Heatmap showing pairwise correlation scores between immune genes and cancer driver genes.

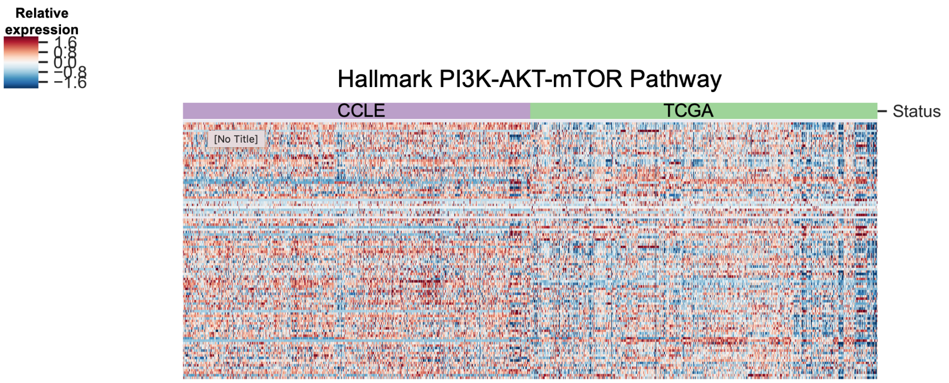

Supplementary Figure 2. Hallmark PI3K-AKT-mTOR Pathway shows non-significant expression change between TCGA and CCLE. Heatmap showing expression of genes in the PI3K-AKT-mTOR pathway in cancer cell lines and tumors. Samples shown are a random subset with equal representation from each dataset in each disease.

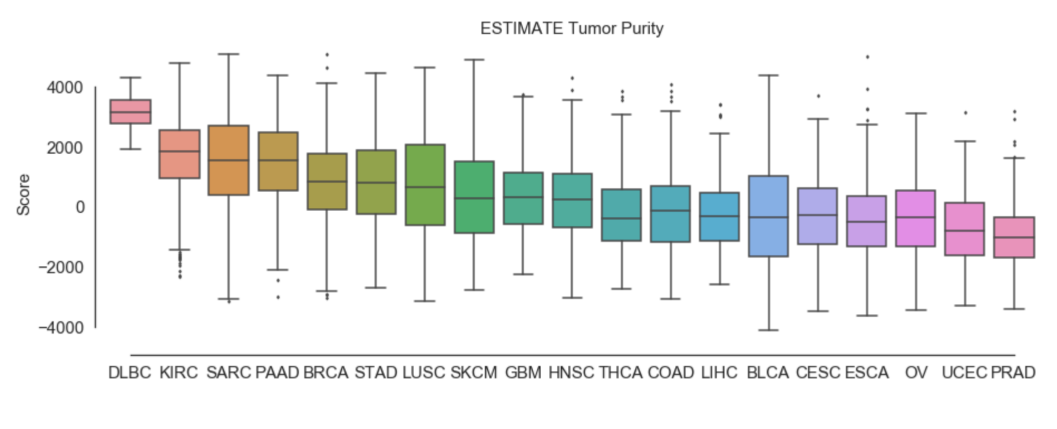

Supplementary Figure 3. ESTIMATE scores for all TCGA tumor samples.

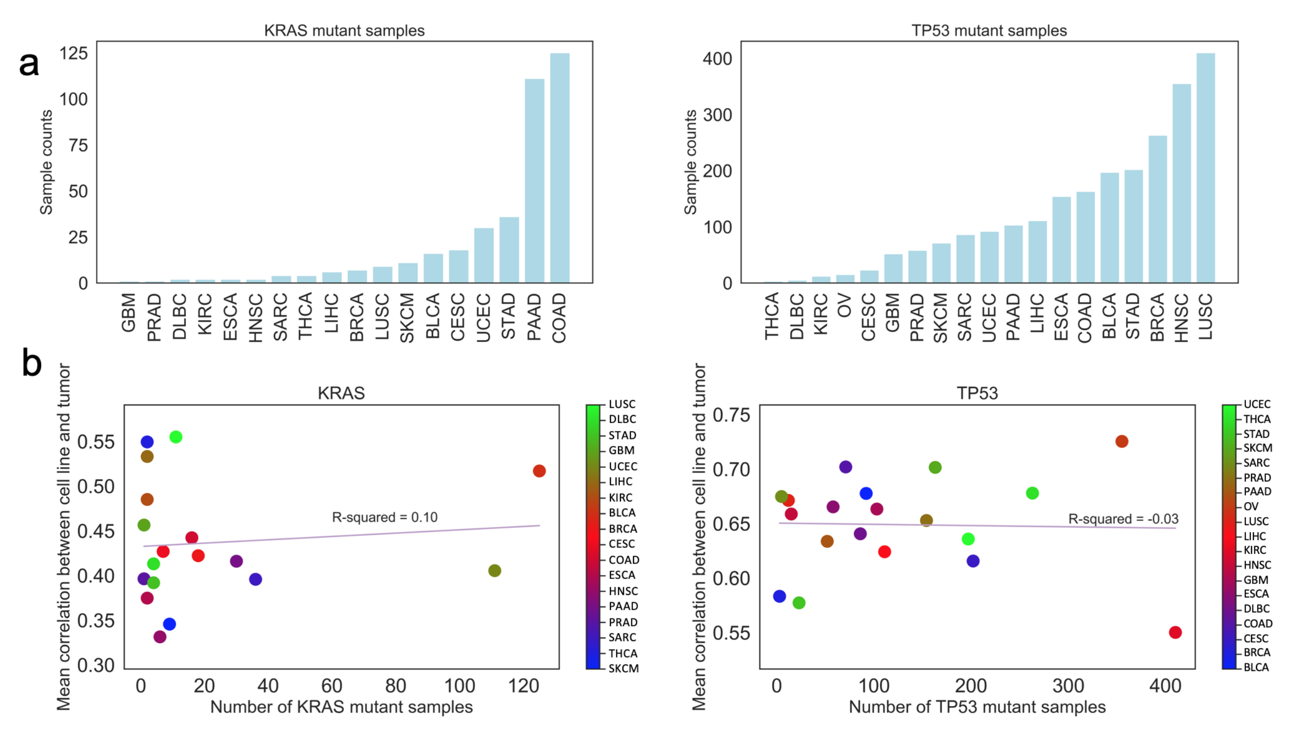

Supplementary Figure 4. Relationship between KRAS or TP53 mutational burden and tumor-cell line correlation by disease type. a) Barplot of KRAS mutant TCGA samples and TP53 mutant TCGA samples by disease type. b) Mean correlation between cell line and tumor in the KRAS pathway (left) and TP53 pathway (right) colored by disease.

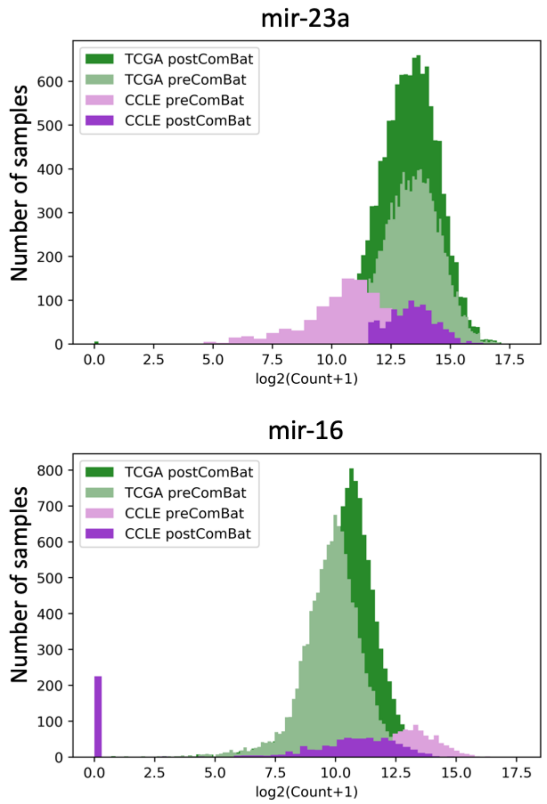

Supplementary Figure 5. Expression of housekeeping miRNA mir-23a and mir-16 pre- and post-ComBat correction in all CCLE or TCGA samples.

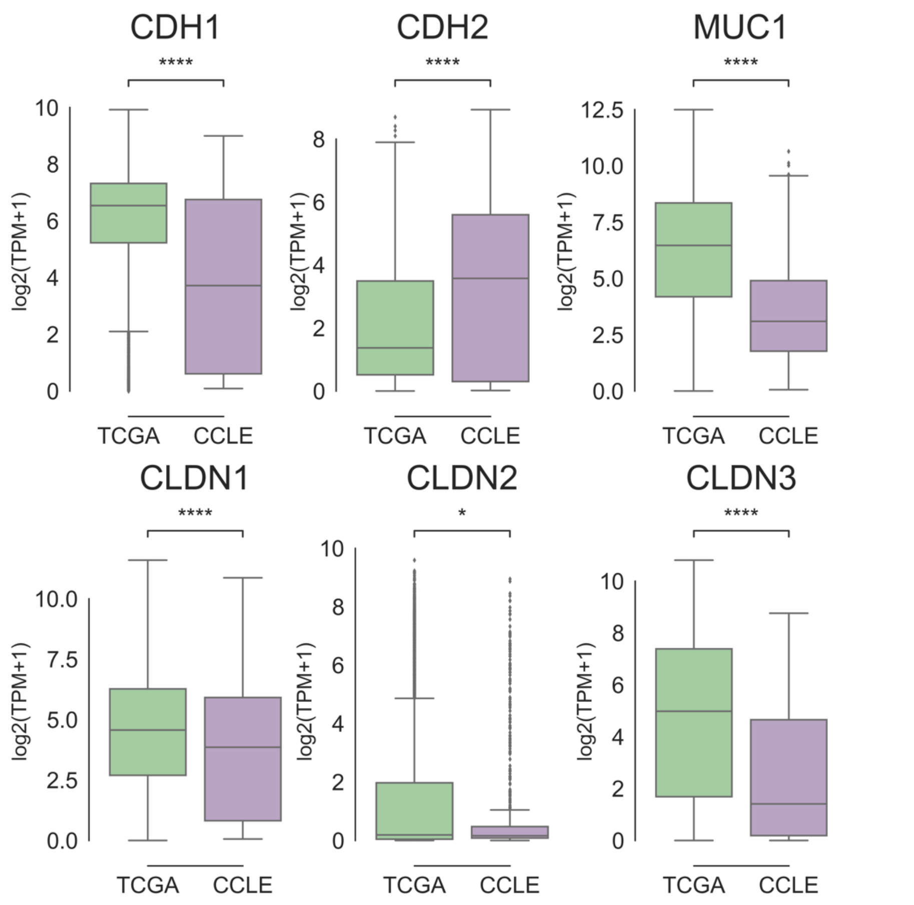

Supplementary Figure 6. Expression of cell type markers in TCGA vs. CCLE. Boxplots showing expression of the E-cadherin/*CDH1* (an epithelial cell marker) and N-cadherin/*CDH2*, *MUC1*, and claudins *CLDN1, CLDN2, CLDN3* (mesenchymal cell markers) in TCGA and CCLE. All samples from both datasets are shown. (Mann-Whitney significance test; * pvalue < 0.05, **** pvalue < 0.0001)

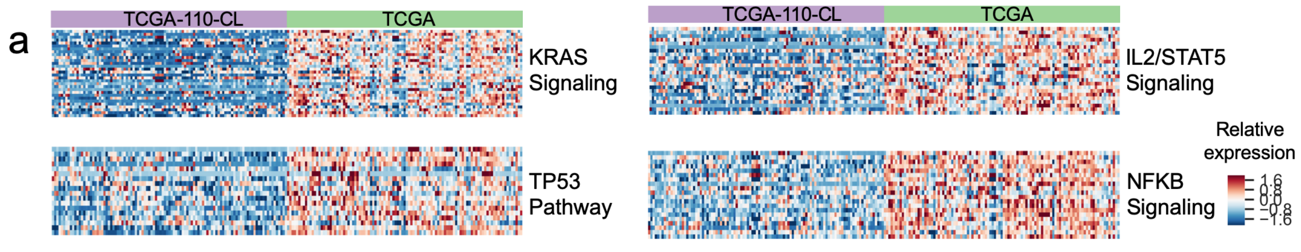

Supplementary Figure 7. Expression of cancer driver pathways in TCGA-110-CL. a) Heatmaps showing expression of SVM-identified genes in 4 cancer driver pathways in TCGA-110-CL cell lines as compared to TCGA samples. Samples shown are a random subset with equal representation from each dataset in each

disease.

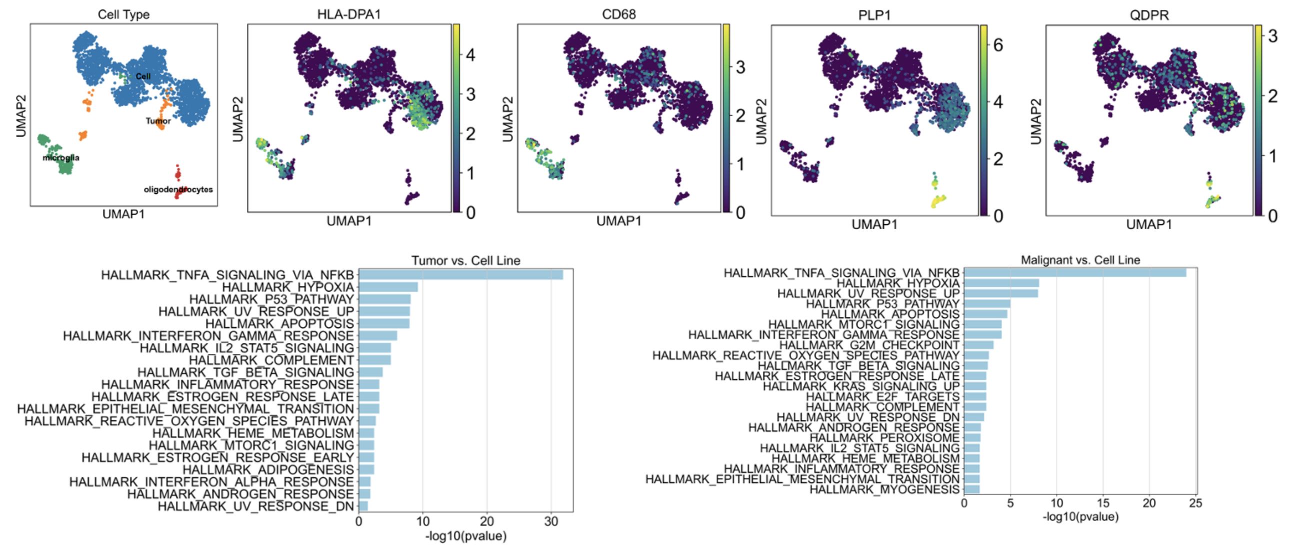

Supplementary Figure 8. Single cell RNA sequencing analysis of matched melanoma metastasis cancer cells and cell culture cells (UAMS Sample CI0000035650). The UMAP plots show cell type markers used to derive cell types. The bar plots show enrichment of cancer pathways in genes with differential expression between either all tumor cells and all cell line cells, or malignant tumor cells and all cell line cells (pvalue < 0.05).

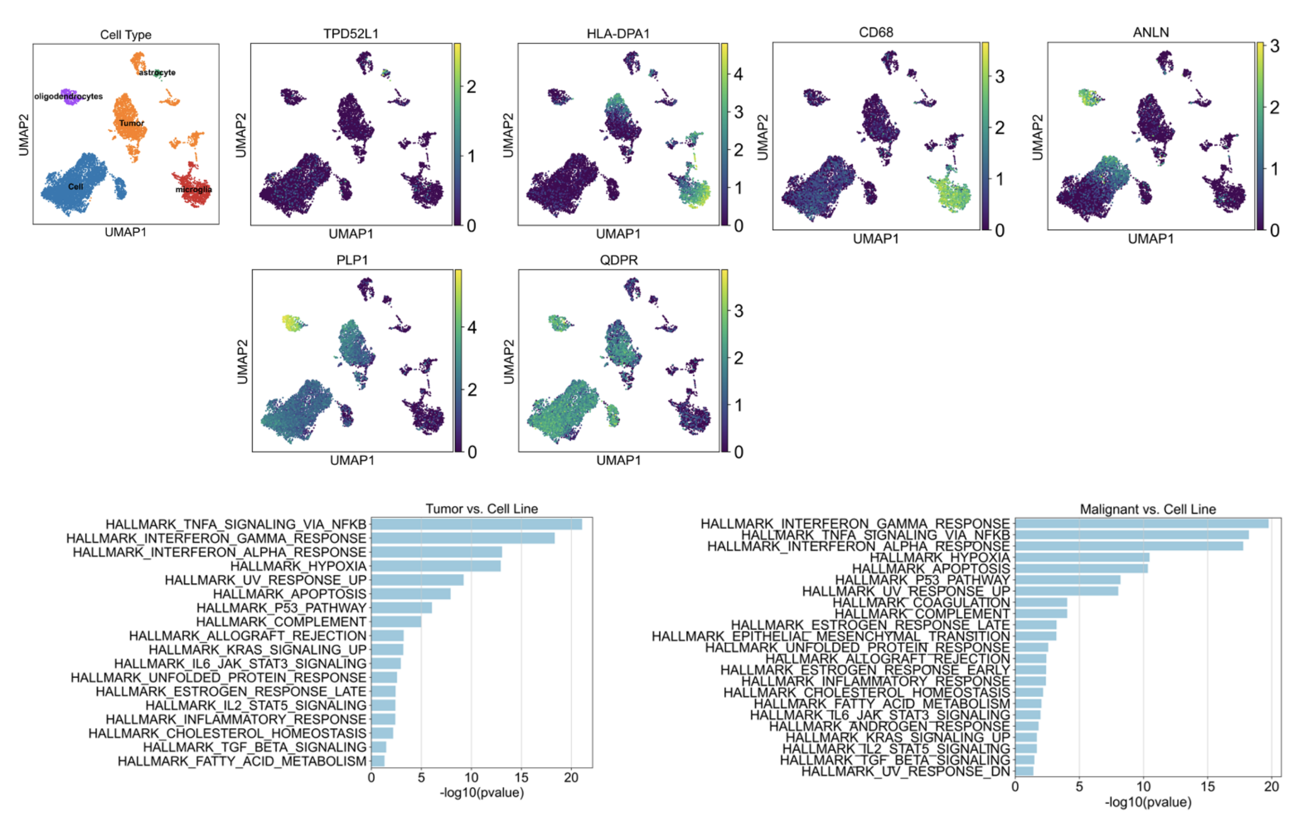

Supplementary Figure 9. Single cell RNA sequencing analysis of matched melanoma brain metastasis cancer cells and cell culture cells (UAMS Sample CI0000035270). The UMAP plots show cell type markers used to derive cell types. The bar plots show enrichment of cancer pathways in genes with differential expression between either all tumor cells and all cell line cells, or malignant tumor cells and all cell line cells (pvalue < 0.05).
