# Supplementary material for: Machine learning multi-omics analysis reveals cancer driver dysregulation in pan-cancer cell lines compared to primary tumors
Source: Commun Biol. 2022 Dec 13;5:1367. doi: 10.1038/s42003-022-04075-4 (PMC9747808; doi:10.1038/s42003-022-04075-4)
Supplement: Supplementary file 3 — Description of Additional Supplementary Files [file 42003_2022_4075_MOESM3_ESM.pdf]

Description of Additional Supplementary Files

File Name: Supplementary Data 1
Description: Top 10% of genes derived from SVM analysis, GSEA results, and results from subset SVM analyses.

File Name: Supplementary Data 2
Description: miRNA, lncRNA and coding gene relationships.

File Name: Supplementary Data 3
Description: Cell type markers and differential expression analysis for single cell data.

File Name: Supplementary Data 4
Description: miRNA and Cytoscape network input.
